# BiGG Models: A platform for integrating, standardizing and sharing genome-scale models

**DOI:** 10.1093/nar/gkv1049

**Published:** 2015-10-17

**Authors:** Zachary A. King, Justin Lu, Andreas Dräger, Philip Miller, Stephen Federowicz, Joshua A. Lerman, Ali Ebrahim, Bernhard O. Palsson, Nathan E. Lewis

**Affiliations:** 1 Department of Bioengineering, University of California, San Diego, 9500 Gilman Drive, La Jolla, CA 92093, USA; 2 Center for Bioinformatics Tuebingen (ZBIT), University of Tuebingen, Tübingen, Germany; 3 Department of Pediatrics, University of California, San Diego, 9500 Gilman Drive, La Jolla, CA 92093, USA; 4 Novo Nordisk Foundation Center for Biosustainability at the University of California, San Diego, La Jolla, CA 92093, USA

## Abstract

Genome-scale metabolic models are mathematically-structured knowledge bases that can be used to predict metabolic pathway usage and growth phenotypes. Furthermore, they can generate and test hypotheses when integrated with experimental data. To maximize the value of these models, centralized repositories of high-quality models must be established, models must adhere to established standards and model components must be linked to relevant databases. Tools for model visualization further enhance their utility. To meet these needs, we present BiGG Models (http://bigg.ucsd.edu), a completely redesigned *Bi*ochemical, *G*enetic and *G*enomic knowledge base. BiGG Models contains more than 75 high-quality, manually-curated genome-scale metabolic models. On the website, users can browse, search and visualize models. BiGG Models connects genome-scale models to genome annotations and external databases. Reaction and metabolite identifiers have been standardized across models to conform to community standards and enable rapid comparison across models. Furthermore, BiGG Models provides a comprehensive application programming interface for accessing BiGG Models with modeling and analysis tools. As a resource for highly curated, standardized and accessible models of metabolism, BiGG Models will facilitate diverse systems biology studies and support knowledge-based analysis of diverse experimental data.

## INTRODUCTION

Biological knowledge bases must evolve to keep pace with the incredible progress in experimental biology. Methods for collecting genome-scale ‘omics’ data have been widely adopted, and the resulting datasets can be difficult to understand, especially when multiple data types are collected in the same experiment (1). These challenges are emblematic of the larger efforts to deal with and capitalize on Big Data (2). A biological knowledge base can serve as a framework for interpreting omics data by providing biological context for each measurement. For this to work, the knowledge base must contain an accurate, genome-scale representation of the organism; it must use unique identifiers and links to existing databases so that scientists can easily align data; and it must describe the relationships between biological networks so that distinct omics data types can be connected during analysis.

Knowledge bases are widely available and commonly used by biologists. The most extensive pathway-oriented knowledge base is the Kyoto Encyclopedia of Genes and Genomes (KEGG) that contains 15 related databases with information on 3982 organisms (3). In contrast, BioCyc is best known for seven highly-curated, multi-scale knowledge bases for model organisms that include *Escherichia coli*, *Bacillus subtilis* and *Homo sapiens* (4). Similar databases are available for model organisms such as yeast (5) and mouse (6). These knowledge bases are all generated through a combination of bioinformatics (e.g. identifying a gene function by sequence homology) and manual curation (e.g. assigning a pathway name to a set of gene products). A complimentary approach is to build a knowledge base around a mathematical model of an organism, and this approach has certain advantages.

Genome-scale metabolic models (GEMs) are mathematically-structured knowledge bases. They contain descriptions of all the biochemical reactions, metabolites and genes in metabolism for a specific organism—a *Bi*ochemical, *G*enetic and *G*enomic (BiGG) knowledge base (7). Additionally, GEMs contain descriptions of the biophysical constraints on metabolic systems (nutrient uptake, oxygen availability, reaction stoichiometry and reversibility) (7). GEMs can be used to predict cellular phenotypes (8), contextualize omics data (9–11), design cell factories (12,13) and understand evolutionary trajectories (14). A further advantage of mathematical structure is that the accuracy of GEMs increases continuously through comparison with experimental data (15).

GEMs have not generally been available through a centralized resource with reliable standards. A workflow for building high-quality GEMs has been described (16), but this process is complex and the quality of published GEMs is highly variable (17). A number of challenges still exist in the reconstruction process. The workflow recommends that metabolites be linked against existing databases (16), but this is not a formal requirement in the models. Visualization of GEMs has been an important feature since the first models were reconstructed, but accessible tools for visualizing GEMs have also been lacking. These challenges have been addressed in the past through unwritten ‘best practices’ in individual labs, but they represent a general challenge when models from different labs are to be collected or compared.

The first BiGG knowledge base was published in 2010, and it addressed some of these challenges for a specific set of 10 GEMs generated at the Systems Biology Research Group at the University of California, San Diego (18). With BiGG, reaction identifiers, metabolite identifiers and pathway maps were formalized in a database, using the software package SimPheny (Genomatica, San Diego CA), and shared on a public website. In BiGG, users could export models in the Systems Biology Markup Language (SBML) format (19), visualize metabolic pathways and search the database. BiGG was a widely-used community resource that was incorporated into other applications (20–23), but it was never extended to be a general resource for storing large numbers of GEMs or for building new GEMs (Table 1). MetRxn is another curated and interactive database of GEMs (23), but it focuses more on identifying metabolite structures and performing model comparisons.

**Table 1.** Comparing BiGG (2010) to BiGG Models (2015)

BiGG (2010)	BiGG Models (2015)
10 models	77 models
Pathway visualization with SVG	Pathway visualization with Escher
Export to SBML Level 2	Export to SBML Level 3, MAT and JSON
	Standardized identifiers for metabolites, reactions and genes
	Public, documented API
	Gene identifiers linked to NCBI RefSeq genome annotation

BiGG Models is a completely redesigned knowledge base that currently includes 77 GEMs linked to 71 genome annotations. It includes a workflow for integrating models built at different times so models can be improved and exported with the latest standards. Model, reaction, metabolite, compartment and gene identifiers are standardized, and pathway maps are included using the Escher pathway visualization library (24). A website allows users to search, browse and visualize the networks. Models can be exported in various community standard formats (25). BiGG Models has a comprehensive application programming interface (API) for accessing and building upon BiGG. With these features, BiGG Models is a platform for integrating, standardizing and sharing knowledge of metabolism.

## KNOWLEDGE BASE CONTENT

BiGG Models is built around a set of high-quality published GEMs. The original models were collected from the supplemental data provided with their publications. Only minimal changes to the models were made (changes are listed in Supplemental Data S6), and the updated models were validated by comparing content and predictions to the published models. These models were aligned in BiGG Models so that they share a common list of reactions and metabolites (‘universal’ reactions and metabolites). Thus, any curation of general attributes like metabolite formulae will apply to all models in the knowledge base, and therefore also provide a standard for future genome-scale metabolic network reconstructions. A total of 77 GEMs are included in BiGG Models as of publication, and more will be added over time.

Genome annotations for the models were downloaded from the National Center for Biotechnology Information (NCBI) reference sequence database (RefSeq) database (26). In total, 71 genome annotations were identified for the GEMs in BiGG Models (a full list of models and genome annotations can be found in Supplemental Data S1).

Pathway maps are included in BiGG Models using the Escher visualization library (24). Maps are currently available for the most widely used models in the database, and more maps are under construction. External database links for metabolites and genes have been included in the database. The external databases include KEGG (3), MetaCyc (4), Reactome (27), HMDB (21), RCSB PDB (28), Model SEED (29) and Entrez Gene (30). Finally, compartment names are often missing from publicly available GEMs, so a list of compartment names was collected in BiGG Models (Supplemental Data S3).

**Figure 1.**
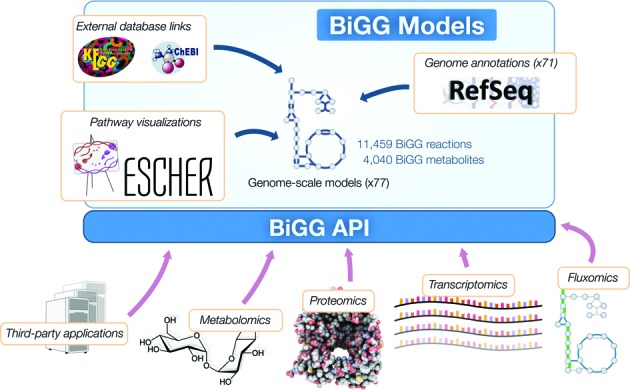
BiGG Models content. BiGG Models is built around a collection of 77 GEMs. The GEMs are integrated into a single database with shared reaction and metabolite identifiers. This core database is enriched with external database links, Escher pathway maps (24), and genome annotations. As a result, BiGG Models is a resource that can be used to analyze and contextualize many omics data types.

BiGG Models integrates these models, genome annotations, pathway maps and additional data in order to provide a set of gold-standard models and a knowledge base of shared biological components (Figure 1). This knowledge base can then be used for analyzing omics data related to reactions (fluxomics), genes (genomics, transcriptomics, proteomics) and metabolites (metabolomics). Recent work has extended GEMs to encompass gene expression (12,31), and eventually these *ME-models* can be included in BiGG, where they can serve as a framework for analyzing protein-associated datasets (proteomics).

## GETTING STARTED WITH BIGG MODELS

### BiGG website

**Figure 2.**
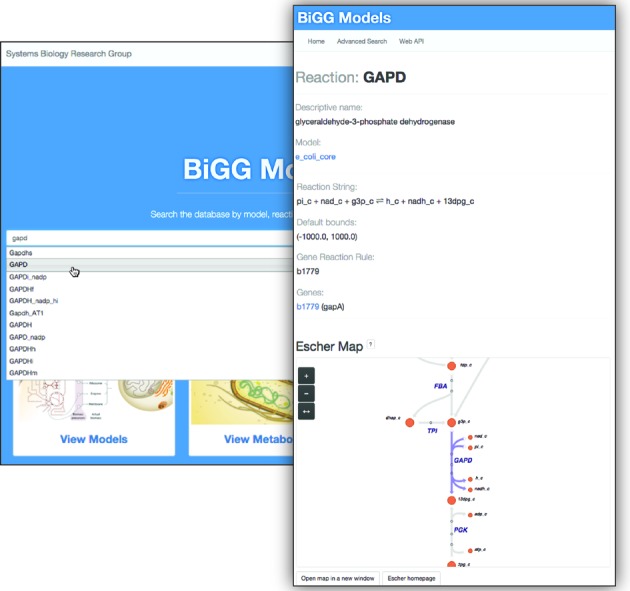
The BiGG Models homepage. The central text box allows users to search for pages in BiGG Models, including models and their reactions, metabolites and genes. Convenient links to the most popular pages about models, metabolites and reactions can be found below the search box. General information about BiGG Models can be found by clicking About at the top of the page.

BiGG Models has a user-friendly website (http://bigg.ucsd.edu) for browsing, searching, visualizing and downloading content. The homepage for BiGG Models includes a search bar for finding models, reactions, metabolites and genes for a search term (Figure 2). It also includes links to lists of all the models, universal metabolites and universal reactions in the knowledge base.

The page for a BiGG model provides an overview of the model and options for downloading the model in community standard formats. The model page also provides a link to the corresponding genome annotation. Reactions and metabolites can be viewed on model-specific pages and universal pages, reflecting the organization of the knowledge base. Model-specific reaction pages include the stoichiometry of the reaction, the reaction bounds within the GEM and the gene-reaction rule for the reaction with links to the related genes. Metabolite pages show the molecular formula for the metabolite and provide external database links. Pages for each gene provide the position of the gene in the chromosome and a link to a page for the genome that contains the chromosome.

The website also includes pathway visualization, advanced search and documentation of the web API. Model, reaction and metabolite pages that have associated pathway visualizations include an embedded, interactive pathway map viewer powered by Escher (an example can be seen on the page http://bigg.ucsd.edu/models/e_coli_core/reactions/GAPD, Figure 2). An **Advanced search** page gives users the options to search for metabolites by external identifier (e.g. KEGG ID) and to find BiGG pages for a specific model. And the **Web API** page has information and examples for using the web API.

### Using BiGG Models for COBRA modeling

The GEMs in BiGG Models can be used for modeling metabolism, interpreting omics data, visualizing metabolic phenotypes and more (9,10,24). BiGG Models makes the models more accessible to users with a variety of options for browsing and downloading them. The GEMs in BiGG Models can be analyzed using the many available *Co*nstraint-*B*ased *R*econstruction and *A*nalysis (COBRA) methods (8,9,32) or any software that reads SBML.

To use a model for COBRA analysis, first download the model in the appropriate format. The most general and most highly annotated format is SBML (SBML Level 3 with Flux balance constraints, an extension of SBML (FBC)), which includes all the content of the model plus the external database links, compartment names and license information. This is the preferred format for analysis in a Python package for COBRA modeling (COBRApy) (32) and the 280+ existing tools can read SBML files (http://sbml.org/SBML_Software_Guide). Models are available in MATLAB MAT format for analysis with the MATLAB COBRA toolbox (33) and the JavaScript Object Notation (JSON) format for building visualizations with Escher (24). With the BiGG Models API, software tools can also access the complete contents of these models programmatically.

### Using BiGG Models for building GEMs

BiGG Models provides a set of identifiers and metabolic components that can be used for new models, as well as a set of standards for defining new IDs (Supplemental Data S4). The BiGG Models API can be used to directly access these identifiers using tools developed for building models.

Using BiGG for building new reconstructions provides a number of benefits. Using BiGG IDs in a new model means that the model can easily be compared to the set of existing models that are already in this knowledge base. The BiGG Models resource is compatible with other tools such as COBRApy (32) and Escher (24), which can also be deployed in other research labs. Thus, using BiGG Models as a guide for new reconstructions will mean that the new reconstruction is compatible with these tools for modeling and visualization. Specifically, the Escher maps in BiGG Models can be adapted to new organisms if the new models utilize the same identifiers (see the Escher documentation for more details at https://escher.readthedocs.org). Finally, BiGG Models can be extended to include models built in other research groups, as long as they conform to the standards set out with BiGG Models.

### Accessing the API

**Figure 3.**
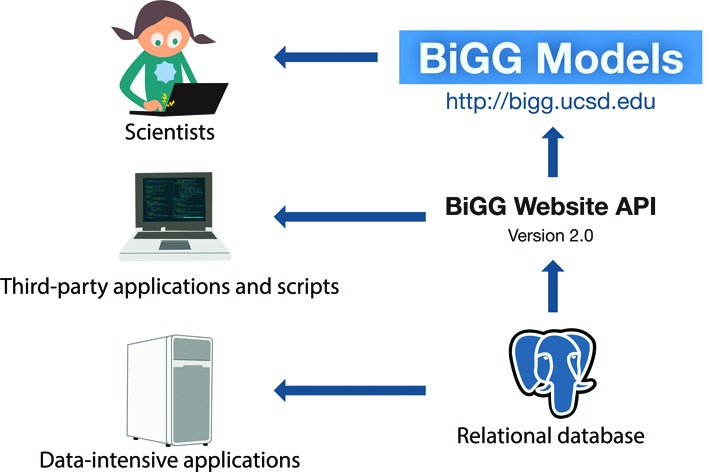
Accessing BiGG. BiGG Models has a user-friendly website for browsing and searching the knowledge base. The knowledge base can also be accessed programmatically using the web API. For more data-intensive applications, it is possible to run a local version of the BiGG database.

BiGG Models includes a fully featured web API (Figure 3). The API can be accessed from any programming language that supports Hypertext Transfer Protocol (HTTP) requests. Thus, BiGG can be used as a service from other applications; for example, a metabolic modeling toolkit could provide direct access to BiGG models *via* the BiGG API. The web API returns JSON formatted data. In the case of an error, an appropriate HTTP error code is returned. The full documentation of the API is provided on the **Web API** page of the BiGG website.

As an example, a list of the models in BiGG Models can be retrieved with the following HTTP request:

GET http://bigg.ucsd.edu/api/v2/models HTTP/1.1

This can be accomplished by visiting http://bigg.ucsd.edu/api/v2/models in a web browser. Many programming languages provide functions for accessing resources on the web. For example, in Python 2.7, the following script will load the data and decode the JSON formatted results:


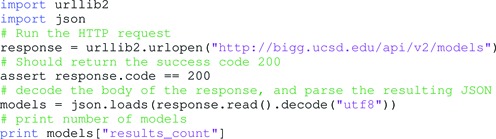


The specific models can then be accessed with follow-up requests. For example, an overview of the first model with BiGG Identifier (ID) e_coli_core can be accessed with a request to the Uniform Resource Locator (URL) http://bigg.ucsd.edu/api/v2/models/e_coli_core, and the full model can be downloaded with a request to the URL http://bigg.ucsd.edu/api/v2/models/e_coli_core/download.

With these tools in hand, developers can use the BiGG API to access any content in the knowledge base from analysis scripts, modeling tools and web applications.

## IMPLEMENTATION OF STANDARDS

### Loading genomes and GEMs

A workflow was developed for integrating models and genome annotations into a single, coherent database. This workflow reconciles any conflicting information, links genes from GEMs to genes in the genome annotations wherever possible and constructs a single database that serves as the basis for BiGG Models. The workflow proceeds as follows. First, a database is initialized in PostgreSQL (PostgreSQL Global Development Group), a high-performance, open-source, relational database. A total of 24 tables are necessary to store the content in BiGG Models (Supplementary Figure S2).

For each genome annotation, genes are loaded into the database with all available identifiers and external database references (Supplementary Figure S5). Genome annotations are used to fill the *genome*, *chromosome*, *genome region* and *gene* tables (Supplementary Figure S2). A single genome can have multiple chromosomes, and genes in each chromosome are loaded from individual files in the Genbank file format (34). The positions of the genes are recorded, and the organism and taxon ID are stored for each genome annotation.

Next, GEMs are loaded into database by reaction, metabolite and gene (Supplementary Figure S5). Efforts are made to separate general information about biological components from model-specific information. The information about reactions and metabolites that is not specific to an organism or a model is considered universal, and BiGG Models represents this information in database tables for universal reactions and universal metabolites. Model-specific information is stored in database tables for model-specific reactions and model-specific metabolites (Supplementary Figure S5). Analogously, information about genes is separated into the annotation-specific gene table in the database and the model-specific gene table. Multiple GEMs may reference a genome annotation; thus, annotation-specific genes can be shared between models.

A further separation is made between metabolites (called *components* in the database tables), which can exist in any cellular compartment, and *compartmentalized metabolites*, which have a specific compartment and participate in reactions.

All the data in BiGG Models that are not found in the GEMs and the genome annotations are arranged in six preference files (Supplemental Data S3).

### BiGG identifiers

BiGG Models uses a set of identifiers—BiGG IDs—that are unique, well-defined, human-readable and memorable (Table 1). BiGG IDs have been used to build GEMs in many research groups, and they were available with BiGG (2010), but problems have appeared with the quality and consistency of BiGG IDs. With BiGG Models, the goal is to provide a single source of correct BiGG IDs that are easy to discover and use in other applications.

**Figure 4.**
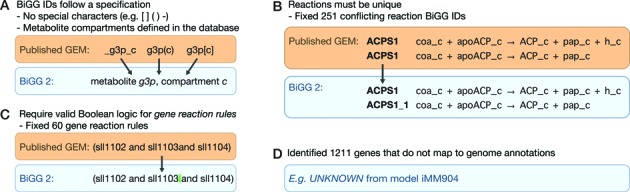
Standardizing GEMs. In order to standardize the GEMs in BiGG Models, a number of changes had to be made to the models. (**A**) First, metabolite and reaction IDs were standardized by removing extraneous characters and using a single format for referring to compartments. (**B**) In cases where the same reaction ID referred to different reactions, one of the reactions received a new identifier. (**C**) Invalid gene reaction rules were manually corrected. (**D**) All the genes that did not map to a genome annotation were recorded for future updates to both the GEMs and the genome annotations.

Now, BiGG IDs follow a simple, clear specification (Supplemental data S4). Reactions, metabolites and genes are assigned unique alphanumeric identifiers, based on the IDs already found in most published GEMs (Figure 4). Metabolites in compartments include a one or two letter compartment code (lowercase letters), and tissue-specific metabolites have a one or two letter tissue code (capital letters). BiGG IDs are now available in the Minimal Information Required in the annotation of Models (MIRIAM) registry with URIs from the identifiers.org service (35) (Table 2).

**Table 2.** BiGG Models contains unique identifiers for models, reactions, metabolites, compartments and genes

Type	Example	BiGG ID	MIRIAM URI
Model	Latest *E. coli* model (36)	*i*JO1366	http://identifiers.org/bigg.model/iJO1366
Reaction	Glyceraldehyde-3-phosphate dehydrogenase	GAPD	http://identifiers.org/bigg.reaction/GAPD
Metabolite	Glyceraldehyde-3-phosphate	g3p	http://identifiers.org/bigg.metabolite/g3p
Compartment	Cytsosol	c	http://identifiers.org/bigg.compartment/c
Gene	*E. coli* gapA	b1779	

For compatibility with existing tools that do not allow numbers at the beginning of identifiers (e.g. SBML), a BiGG ID can be prefixed with R_ for reactions and M_ for metabolites. Unprefixed BiGG IDs are used on the BiGG website, in Escher and in COBRApy, and prefixed BiGG IDs are automatically generated in exported SBML files.

Genes in BiGG Models have identifiers that are unique to a specific genome annotation. Thus, genes are referenced by their locus IDs in the genome annotation. Genes that do not map to a genome annotation retain the ID from the original model file. Gene BiGG IDs are prefixed with G_ in exported SBML files, and unprefixed gene IDs are used in BiGG Models, Escher and COBRApy.

### ModelPolisher

BiGG Models supports the latest SBML standard Level 3 Version 1 with FBC version 2 (37). To generate compliant and highly-annotated files, the ModelPolisher application was developed (https://github.com/SBRG/ModelPolisher). SBML models are first generated using COBRApy (32), then ModelPolisher inserts MIRIAM annotations and adds specific terms from the Systems Biology Ontology (SBO) (38) to individual model components in order to better point out their role. The SBO is a collection of controlled vocabulary terms with clear definitions and references. For the annotation of BiGG models, the following new terms have been added to SBO: flux bound (SBO 625, SBO 626), exchange reaction (SBO 627), demand reaction (SBO 628), biomass reaction (SBO 629) and ATP maintenance (SBO 630). The resulting SBML files are available on the model pages of the BiGG Models website, and an overview of the model content can be seen by loading a downloaded SBML file in a web browser.

### Design and implementation

BiGG Models is a modular application composed of a relational database, a web API and a website (Figure 3). It is primarily written in Python 2.7, SQL, JavaScript, HTML and CSS.

BiGG Models is built with PostgreSQL 9.4.4 (PostgreSQL Global Development Group, http://www.postgresql.org/). The SQLAlchemy (http://www.sqlalchemy.org/) object relational mapper is used to load and query from the database. The website and API servers are implemented with Tornado (http://www.tornadoweb.org/en/stable/). The website retrieves data through the same web API provided to users; thus, all the content in the website is available through the API. A number of other libraries were essential for building BiGG Models, including Jinja2 (http://jinja.pocoo.org/), JQuery (https://jquery.com) and TableSorter (https://mottie.github.io/tablesorter/docs/).

## CONCLUSION

BiGG Models is a fully redesigned platform for integrating, standardizing and sharing GEMs of metabolism. The knowledge base currently integrates the metabolic content from 77 GEMs and 71 genome annotations, and users can search and explore the knowledge base with the BiGG website. A web API is available for building new applications that extend the capabilities of BiGG. The result of these features is a knowledge base that can be used to understand a huge variety of experimental data.

BiGG is free for academic and non-profit use so that the community can easily use and extend the knowledge base. The BiGG Models source code is available on GitHub (https://github.com/SBRG/bigg_models). If a user finds a model error or a website bug using the BiGG Models website, it is possible to submit a report to the maintainers so this issue can be resolved. Each page for a reaction, gene, metabolite or model includes a form to submit such a report, along with instructions. Website bugs can be fixed with future software releases. Model issues, in contrast, cannot be immediately fixed because BiGG is meant to present GEMs that are mathematically equivalent to the published models (though identifiers have been modified). Therefore, model issues will be collected for future updates to the GEM for that organism.

BiGG Models will continue to be developed to meet the needs of experimental and computational biologists. New visualization tools and model analysis features are in the works. The next generation of models can eventually be included in BiGG; these models incorporate expression networks, increased spatial resolution, regulation and protein structures into GEMs (8,12,31). Plans for future BiGG releases will be driven by ongoing feedback from the users of the BiGG Models knowledge base.

## AVAILABILITY AND REQUIREMENTS

BiGG Models is freely available online for academic and non-profit use at http://bigg.ucsd.edu, and a JavaScript-enabled browser is required to access certain features. The requirements for viewing Escher maps can be found on the Escher website (https://escher.github.io). Installation of an independent system requires Python 2.7 and PostgreSQL 9.4.4 or later.

## SUPPLEMENTARY DATA

Supplementary Data are available at NAR Online.
